# Data on mitigation policies at local level within the Covenant of Mayors’ monitoring emission inventories

**DOI:** 10.1016/j.dib.2020.106217

**Published:** 2020-08-24

**Authors:** Valentina Palermo, Paolo Bertoldi, Malvina Apostolou, Albana Kona, Silvia Rivas

**Affiliations:** a European Commission, Joint Research Centre, Via Enrico Fermi 2749, I, Ispra VA 21027, Italy; b UMR MAP-MAACC 3495 CNRS/MCC, École Nationale Supérieure d'Architecture de Paris-La Villette, France

**Keywords:** Covenant of Mayors, Monitoring emission inventory, Local authorities, Mitigation policies, Governance, CO_2_ reduction

## Abstract

This data article relates to and complement the research paper: "Assessment of climate change mitigation policies in 315 cities in the Covenant of Mayors initiative" [1]. The reported data has been collected and elaborated within the framework of the Covenant of Mayors (CoM) initiative. The dataset is extracted from the overall database of the initiative reported through the platform *MyCovenant.* The data deals with the Monitoring Emission Inventories submitted by local authorities by 2016. Data has been processed and elaborated to highlights specific features of signatories and the policies they have adopted in the development of their Sustainable Energy Action Plans (SEAP). Available data relates to the mitigation policies in the SEAPs and includes their carbon reduction potential, the status of implementation and the class of governance. The CoM gathers together thousands of local authorities who voluntary committed to decarbonisation and increase resilience in their territory. Therefore, this data can be of interest for local policy makers and urban planners to identify successful examples in cities with comparable contexts and to identify possible measures in several sectors for climate change mitigation.

**Specifications Table**

**Table:** 

**Subject**	Geography, Planning and Development
**Specific subject area**	Climate change mitigation policies at local level
**Type of data**	Datasheet Table Numeric Text
**How data were acquired**	Data is submitted by local authorities through *MyCovenant* reporting platform
**Data format**	Raw analysed .xls
**Parameters for data collection**	Data regarding signatories and mitigation policies is obtained from local authorities participating in the Covenant of Mayors initiative and submitting their Monitoring Emission Inventories. Data regarding assigned governance are obtained from the analysis developed in [1], GDP information are extrapolated from Eurostat and Heat Degree Days from [5].
**Description of data collection**	Signatories to the Covenant of Mayors report on their strategies and actions included in their Sustainable Energy Action Plans (SEAPs) through the online platform: MyCovenant
**Data source location**	Local Authorities who have signed the Covenant of Mayors Initiative and submitted their Monitoring Emission Inventory by 2016 (both in EU and Non EU countries)
**Data accessibility**	With the article In the process of being included in the Joint Research centre Data Catalogue with the whole CoM dataset, available at: https://data.jrc.ec.europa.eu/collection/id-00172
Related research article	V. Palermo, P. Bertoldi, M. Apostolou, A. Kona, S. Rivas, Assessment of climate change mitigation policies in 315 cities in the Covenant of Mayors initiative, Sustainable Cities and Society. 60 (2020) 102,258. https://doi.org/10.1016/j.scs.2020.102258.

**Value of the Data**

The data gives quantitative and qualitative information on the local mitigation policies implemented to achieve the 20% carbon reduction target by local authorities which are part of the Covenant of Mayors up to 2016
Climate change research, local government, policy makers, urban science are the fields where this data provides valuable information.
Data can be used for comparisons with other data on local policies, for investigation on climate change mitigation and adaptation, for further analysis. Urban planners and local policy makers can benefit from the data
The data supports regular evaluation of the carbon reduction target within the CoM initiative and the sharing of successful approaches and methodologies.
The data gives insight on mitigation policies in different sectors adopted in local authorities across Europe.

## 1 Data description

Cities play a key role in the climate challenge and are the place where local experimental governance aiming at meeting low carbon objectives can be tested [2]. The EU Covenant of Mayors (CoM) is an international initiative, part of the Global Covenant of Mayors, that directly engages local governments to adopt climate and energy targets at least matching the EU targets. By voluntarily adhering to the CoM, local authorities committed to decarbonise and increase resilience in their territory and share their emission inventories and climate action plans [3]. Up to 2015 the CoM commitment was to achieve and exceed by 2020 at least the European 20% reduction of the total emissions objective compared to the baseline, in the area of influence of the local authority, by the implementation of a Sustainable Energy Action Plan (SEAP) [4]. In addition, signatories commit to preparing and submitting an implementation report and a Monitoring Emission Inventory (MEI), which allows appraising the progresses in achieving their target. The data, included in the datasets in the supplementary data, includes 12 307 mitigation policies adopted by 315 local authorities, which submitted their first Monitoring Emission report by 2016. Those 315 local authorities are considered CoM pioneers cities, since they have been able to report their MEI by 2016. Data has been collected from the MEIs, processed and cross related with information regarding mode of Governance as introduced in [2] and developed in [1], Gross Domestic Product (GDP) and Heat Degree Days (HDD) and are reported in a spreadsheet. Data is organised in two datasets, which have been built by merging information regarding the signatories and the mitigation policies with the other variables. The two datasets included in the supplementary material are:

1- “Signatories”: the information on local authorities (country, climate characteristics, Gross Domestic Product expressed as Purchasing Power Standard – PPS and the number of policies per mode of governance)
2- “Policies” the mitigation policies included in the MEIs submitted by local authorities, with specification on sectors (such as: buildings, transport, public lighting, etc.), mode of governance, the corresponding carbon reduction potential (tCO_2_), the responsible local organisation for the implementation and the status of implementation.

In particular, the variables included in the dataset are:

**Table:** 

MUNI CODE	A unique code to identify the signatory
Country Code	The country where the local authority is located according to NUTS coding and nomenclature
N. Inhabit	The number of inhabitants of the local authority as reported by 2016
Latitude	Geographic coordinate in WGS84 system
Longitude	Geographic coordinate in WGS84 system
Climate Code	The code refers to three intervals of Heating Degree Days of local authorities
GDP	The gross domestic product of the country where the local authority is located
BEI	The amount of tons of carbon dioxide as reported in the baseline emission inventory of the local authorities
BEI_INH	The number of inhabitants as reported in the baseline emission inventory of the local authorities
MEI	The amount of tons of carbon dioxide as reported in the Monitoring emission inventory of the local authorities
MEI_INH	The number of inhabitants as reported in the monitoring emission inventory of the local authorities
GDP NUT3	The gross domestic product of the area at NUTS 3 level, where the local authority is located
N policies aggregated	Number of policies planned in the SEAPs and reported via MyCovenant platform by the local authority
N-Education Aggregated	Number of policies planned in the SEAPs and reported via MyCovenant platform by the local authority belonging to the Education mode of governance
N-Municipal self governing aggregated	Number of policies planned in the SEAPs and reported via MyCovenant platform by the local authority belonging to the Municipal self governing mode of governance
N-Financing Aggregated	Number of policies planned in the SEAPs and reported via MyCovenant platform by the local authority belonging to the Financing and provision mode of governance
N-Regulation Aggregated	Number of policies planned in the SEAPs and reported via MyCovenant platform by the local authority belonging to the Regulation mode of governance
N-Other Aggregated	Number of policies planned in the SEAPs and reported via MyCovenant platform by the local authority belonging to the Other mode of governance
ID ACTION	The unique code for identifying the action
Sector	The sector the action refers to
key action description	The action description
Area_of_intervention	The area of intervention of the action
Policy_Type	The related policy type
Origin of the action	The origin (responsible body) of the action
Status of implementation	The status of implementation of the action at the moment of submission: completed, new, not started, postponed and ongoing
Governance	The type of governance the action refers to
CO2 red	The amount of tCO_2_ reduction the planned action contributes to

## 2 Experimental design, materials, and methods

The experimental design of the study is to relate the local authorities’ characteristics with their planned mitigation policies and the governance approach. Detailed studies and investigation on multi-level governance and policies were previously developed and investigated to base the structure of the analysis [5,6]. The dataset included as supplementary material has been developed from the information submitted by local authorities, which signed the CoM and reported through the MyCovenant official platform [7]. The data extracted from the platform focused on the sample of local authorities, which submitted a MEI by 2016 (315 local authorities) and on their mitigation policies (12 307). Therefore, the present dataset is the first of its kind to deal with MEIs and urban governance. Further information was collected and added to the database (GDP, climate characteristics and governance) [1,8,9]. In particular, the datasheet “Policies” includes fields, which specify the policies characteristics (sectors, responsible organisation, description, status of implementation) and assign them to a specific type of governance. This is implemented according to the aggregation procedure developed in [1], in which the fields of area of intervention and policy type are related to different modes of governance as firstly developed by [2]. The indicator used to define the climatic characteristic of a local authority is the Heating Degree Day (HDD) index, a weather-based technical index designed to describe the need for the heating energy requirements of buildings, it is aggregated and presented on NUTS-2 level, and NUTS-3 level [10]. The climate characteristics of the local authorities are expressed using a code that goes from "1″ to "3″, where "1″ refers to cold climate conditions, "2″ intermediate and "3″ warm [8,10]. This allows, on one hand to increase the information and build figures related to the local authorities’ features and on the other, to associate a mode of governance to the policies planned by local authorities. The data were evaluated by examining their consistency to improve readability and allow cross-relating the mitigation measures to other factors such as sectors, climate [8,10], GDP [9,11], number of inhabitants, country. The dataset can be used as supporting tool for policy–makers and urban planners dedicated to urban low-carbon development, it provides a database for existing mitigation policies at local level and it may facilitate the identification of best-practice examples and replicable methodologies in similar urban contexts.

## Declaration of Competing Interest

The authors declare that they have no known competing financial interests or personal relationships that could have appeared to influence the work reported in this paper.

## Appendix Supplementary materials

Image, application 1
